# Social functioning profiles, attention skills and learning: what relationships in early childhood?

**DOI:** 10.3389/fpsyg.2026.1819472

**Published:** 2026-06-02

**Authors:** Elena Commodari, Francesca Foti, Valentina Lucia La Rosa

**Affiliations:** Department of Educational Sciences, University of Catania, Catania, Italy

**Keywords:** attention, early childhood, early learning, learning abilities, preschool children, social functioning profiles

## Abstract

**Background:**

Early learning emerges from the interplay between cognitive processes and social experiences. While attention is widely recognized as a core foundation of preschool learning, less is known about whether distinct aspects of attention contribute uniformly to learning outcomes or whether their functional relevance varies across children’s social functioning profiles.

**Methods:**

The study involved 139 preschool children (4–5 years). Multiple aspects of attention (reaction times, simple and related to a choice; focused attention; short-term span of attention; divided and alternating attention) were assessed using a computerized battery. Learning abilities were evaluated through an observational questionnaire measuring general learning abilities, prerequisites for literacy and mathematics, and specific domains of learning performance. Social functioning profiles were assessed using an observational Q-sort procedure. Hierarchical regression models were estimated to examine independent associations between attentional aspects and learning outcomes, and moderation analyses tested whether social functioning conditioned these associations.

**Results:**

Visual focused attention, visual–spatial focused attention, and short-term span showed consistent positive associations with global and domain-specific learning outcomes. Divided attention also contributed to learning performance, but its association varied systematically as a function of social functioning profiles, being significant at mean and high levels but not at low levels, consistently across general learning abilities and domain-specific prerequisites.

**Conclusion:**

Findings suggest that early learning competence reflects not only the internal architecture of attention, but also the social contexts in which specific aspects of attention are concurrently expressed. While several components of attention show stable associations with learning outcomes regardless of relational context, the contribution of divided attention is contingent on children’s social functioning profiles within adult-child interactions.

## 1 Introduction

### 1.1 Social functioning profiles in early educational contexts

Social functioning profiles refer to relatively stable patterns through which children organize their behavioral engagement within interactions with adult caregivers, encompassing cooperation, emotional availability, responsiveness to guidance, and orientation toward social partners as reference points for exploration and task engagement (Cassibba et al., 2000). Rather than describing isolated relational behaviors, these profiles capture children’s broader behavioral organization within adult-child interactions, reflecting how they respond to social cues and situational demands across educational contexts (Sroufe, 2005; Thompson, 2008). As a construct, social functioning profiles draw on and integrate contributions from multiple theoretical frameworks. From attachment theory, they incorporate the emphasis on the quality and organization of children’s behavioral responses within specific caregiver relationships (Sroufe, 2005; Thompson, 2008). From self-regulation research, they reflect the role of adult-child interaction quality in scaffolding children’s behavioral organization and effortful engagement within structured contexts (Blair and Raver, 2015). From developmental systems approaches, they embody the premise that children’s relational behavioral patterns are dynamically expressed within specific contextual configurations rather than constituting fixed individual traits (Kärtner and Köster, 2024). While these frameworks each illuminate important dimensions of children’s relational functioning, the conceptualization of social functioning profiles is not reducible to any single one, but they describe context-specific and relationship-specific patterns of behavioral organization within adult-child interactions, capturing dimensions that span the relational, regulatory, and interactional levels of children’s engagement with non-parental caregivers.

Beyond the family environment, early educational settings represent a central context in which social functioning profiles are expressed and consolidated. In these settings, children are increasingly required to interact with non-parental adults, coordinating their behavior with adult expectations, participate in structured group routines, and follow externally guided activities (Bergin and Bergin, 2009; Birch and Ladd, 1997; Commodari, 2013a, 2013b). Empirical evidence indicates that children displaying more adaptive social functioning patterns show stronger socio-emotional adjustment, greater engagement in educational activities, and more adaptive learning-related behaviors (Bergin and Bergin, 2009; Birch and Ladd, 1997; Pianta, 1999). Recent research further highlights the specific role of emotional processes within these relational dynamics. Educator-child relationships characterized by emotional sensitivity, warmth, and responsiveness have been associated with stronger emotional self-regulation and inhibitory control in preschool children (Hatfield et al., 2022), and a meta-analysis of 57 studies found that the emotional dimensions of self-regulation show significant positive associations with children’s social competencies during early childhood (Geng et al., 2024). These findings underscore that emotional availability, one of the defining dimensions of adaptive social functioning profiles, is not merely a relational quality but a functionally relevant feature of the interactional context that may support children’s engagement in cognitively demanding activities (Hamre and Pianta, 2003).

Contemporary developmental models conceptualize social functioning and cognitive development as dynamically intertwined processes (Kärtner and Köster, 2024). During the preschool years, children’s daily experiences increasingly involve participation in shared activities with non-familial adults, where they are required to coordinate their behavior with external expectations and situational demands. These interactional contexts are associated with growing demands on the cognitive functions that support engagement, behavioral organization, and responsiveness to guidance. Among these functions, attention occupies a central position, as it enables children to orient toward relevant cues, sustain engagement, and manage competing sources of stimulation (Mahone and Schneider, 2012). In this developmental phase, understanding children’s social functioning becomes essential for clarifying how relational experiences may be associated with the development of attentional processes that support early learning.

### 1.2 Attention as a cognitive process embedded in social contexts

Attention can be defined as a set of neurocognitive processes that enable individuals to achieve and maintain an adequate level of alertness, orient toward relevant information, and regulate cognitive and behavioral responses in a goal-directed manner (Petersen and Posner, 2012; Posner and Rothbart, 2014). In early childhood, attention plays a central role in supporting engagement with structured activities, as it allows children to manage incoming stimulation, follow external guidance, and adapt behavior to situational demands (Commodari et al., 2025).

From a functional perspective, attention should not be conceived as a unitary capacity, but rather as a multifaceted system composed of several functionally distinct aspects that mature gradually across childhood. Among these key aspects, we can distinguish focused, divided, and alternating attention (Commodari, 2017). *Focused attention* involves the capacity to selectively concentrate on task-relevant information while inhibiting interference from competing or distracting stimuli (*selective attention*), thereby facilitating efficient detection and processing of environmental inputs (Cohen, 2014). *Divided attention* refers to the ability to process multiple sources of information simultaneously, whereas *alternating attention* allows individuals to flexibly shift between tasks or cognitive sets (Hirano et al., 2020; Rueda, 2014). At a more fundamental level, early attentional functioning also encompasses processes related to *alertness and general physiological activation*, which support readiness to respond to environmental demands, and are commonly measured through simple reaction time paradigms (Cohen, 2014; Rueda, 2014). Finally, effective attentional functioning relies on the ability to temporarily maintain attentional representations across sequential inputs (*short-term span of attention*), which supports the coordination and allocation of attentional resources during goal-directed activities. Attention span refers to the number of objects or units of information that an individual can clearly perceive and retain simultaneously, reflecting the extent of immediate perceptual and mnemonic capacity at a given moment (Lanyu, 2024). Together, these functionally distinct aspects constitute the attentional architecture that underlies children’s engagement with the environment across development.

Specifically, between three and 5 years of age, children show substantial developmental gains in endogenous attentional control, including improvements in focused, divided, and alternating attention (Graziano et al., 2011; Raver et al., 2012). Importantly, the development of attentional processes during this period does not occur in isolation from children’s relational experiences. Everyday interactions with adults provide structured expectations, feedback, and shared activity frameworks that influence how different key aspects of attention are coordinated and expressed in behavior (Blair and Raver, 2015; Casagrande et al., 2021).

From a developmental systems perspective, cognitive processes and social experiences do not unfold in parallel but are mutually constitutive across development (Kärtner and Köster, 2024; Thelen and Smith, 1994). Within this framework, attentional functioning in early childhood is not a fixed property of the individual cognitive system, but a capacity whose expression is dynamically associated with the relational and contextual structures in which children participate. Social functioning profiles within caregiver relationships represent precisely the kind of stable contextual condition that, according to developmental systems models, is associated with differences in how internal cognitive resources are expressed in observable behavior.

Importantly, not all components of attention may be equally sensitive to this relational context. Self-regulation research consistently shows that the development of effortful control—the volitional modulation of attention and behavior in goal-directed contexts—is scaffolded by the quality and predictability of adult-child interactions (Blair and Raver, 2015; Raver et al., 2012). Therefore, components of attention that require the coordination of multiple concurrent demands within structured interactional contexts may be more contingent on the relational organization that social functioning profiles provide, compared to components primarily reflecting the efficiency of internal processing resources. Evidence further indicates that more adaptive relational patterns with non-parental caregivers are associated with stronger attentional regulation and greater flexibility in allocating cognitive resources across structured activity contexts (Pallini et al., 2019; Pasco Fearon and Belsky, 2004). Therefore, attention represents one of the primary mechanisms through which social functioning profiles may become functionally linked to early learning performance, and the extent to which this association operates differentially across distinct attentional components remains an open empirical question that the present study aims to address.

### 1.3 Attention and early learning abilities

During the preschool years, attention constitutes a core cognitive foundation for early learning abilities. As children transition toward increasingly structured routines, they must sustain engagement, follow instructions, coordinate multiple sources of information, and flexibly adapt their responses to task demand (Blair and Raver, 2015). Attention enables children to select, maintain, and integrate learning-relevant information, thereby supporting their participation in and playful educational activities (Posner and Rothbart, 2014).

Distinct key aspects of attention contribute differentially to early learning. Focused attention supports the processing of relevant sensory input and inhibition of competing stimuli, processes that are particularly relevant for phonological discrimination, language comprehension, and symbolic processing (Commodari, 2017; Franceschini et al., 2012). Divided and alternating attention permit the coordination of multiple operations, such as integrating auditory and visual information or shifting between representational systems, which are central to the acquisition of complex behavioral competencies, early literacy, and numeracy development (Commodari et al., 2025).

Empirical findings consistently indicate that children who demonstrate more efficient attentional functioning exhibit stronger play skills and better performance in emergent literacy, early mathematics, and broader indicators of learning readiness (McClelland et al., 2007). Conversely, weaknesses in attentional processes have been associated with less stable learning trajectories and increased risk for later academic difficulties, even in the absence of clinical diagnoses.

In early childhood, the main aspects of attention develop within relational contexts. Adaptive social functioning may facilitate sustained engagement and flexible attentional allocation in response to adult cues, whereas less adaptive profiles may constrain the effective translation of attentional resources into observable learning performance (Pallini et al., 2019; Pasco Fearon and Belsky, 2004). Attention may therefore serve as a functional bridge linking social functioning profiles in adult-child interactions to emerging learning competencies.

### 1.4 Research gap and study objectives

Although extensive research has documented the importance of social experiences and attentional processes in early development (Kärtner and Köster, 2024), evidence remains fragmented regarding how children’s social functioning profiles interact with specific aspects of attention in relation to early learning abilities. Previous studies have typically examined relational experiences and attention separately or focused on the contribution of attention to learning outcomes without considering relational variability (Commodari, 2012, 2013a, 2013b).

Moreover, relational dimensions have often been conceptualized in broad terms, such as global indicators of classroom climate or relationship quality in the educational settings (Bergin and Bergin, 2009; Birch and Ladd, 1997; Hamre and Pianta, 2003), rather than examining how children’s observable social functioning profiles may condition the association between distinct aspects of attention and learning performance. Given that attentional demands in preschooler are embedded within adult-guided activities, it is plausible that the strength of the association between specific aspects of attention and learning outcomes varies as a function of children’s social functioning profiles.

In light of these considerations, the present study adopts a conditional modeling approach to examine whether social functioning profiles moderate the association between key aspects of attention and early learning abilities in preschool children. Specifically, the study aims to: (1) examine differences in the functioning of key aspects of attention and learning abilities across levels of social functioning; (2) investigate the independent contributions of different aspects of attention to learning outcomes; and (3) test whether the associations between specific aspects of attention and learning abilities vary as a function of children’s social functioning profiles within adult-child interactions.

Through this integrative framework, the study aims to clarify whether social functioning conditions the extent to which the main aspects of attention are associated with children’s emerging learning competencies during a period of heightened developmental plasticity.

## 2 Materials and methods

### 2.1 Participants and setting

This study involved preschool-aged children between 4 and 5 years of age attending two public early childhood education centers located in an Italian municipality. Within the Italian educational framework, early childhood education represents a stage that spans from 3 to 5 years of age and precedes entry into primary school at 6 years. Accordingly, all participating children were enrolled in the final phase of preschool, a period characterized by increasing exposure to structured activities and learning-related routines that anticipate, without fully overlapping with, the formal demands of primary education.

During this stage, children are supported in the development of foundational competencies relevant for later academic learning, such as language comprehension and early symbolic skills, while formal reading and writing instruction has not yet begun. Eligibility criteria required that children had attended the same preschool setting for at least one full academic year and were enrolled for a minimum of 25 h per week. Children with diagnosed neurodevelopmental disorders, sensory impairments, or other conditions likely to interfere with cognitive or behavioral functioning were excluded from participation.

The final sample consisted of 139 children (71 boys and 68 girls). Seventy children were 4 years old and sixty-nine were 5 years old (*M* = 4.50, *SD* = 0.50). The sample included children of Italian (*n* = 103) and non-Italian nationality (*n* = 36). All participants came from families in which Italian was the primary language used in daily communication. This included families of foreign origin in which both parents had resided in Italy for several years and reported fluent use of Italian.

### 2.2 Measures

This study used three assessment tools to examine the key aspects of attention, social functioning profiles, and learning abilities in preschool children. Attention was assessed using a computerized battery designed to evaluate the different aspects in which attention is articulated (Di Nuovo, 2000, 2024). Social functioning profiles were assessed through an observational Q-sort methodology that captures children’s characteristic patterns of behavior, regulation, and engagement within everyday educational interactions (Italian version by Cassibba and D’Odorico, 2000; original framework by Waters, 1995). Learning abilities were evaluated using an observational questionnaire aimed at providing a global index of children’s ability to learn, together with domain-specific indicators of general learning-related skills and foundational prerequisites for literacy and mathematics learning.

The *Attention and Concentration Battery* (Di Nuovo, 2000, 2024) is a computerized assessment composed of several tasks designed to evaluate the key aspects of attention in different ages, including preschool-aged children.

*Focused attention* was assessed through modality-specific recognition tasks in which children were required to respond to predefined target stimuli presented in auditory, visual, or visual–spatial formats, while ignoring distractors. *Divided attention* was evaluated using a dual-task paradigm in which children were asked to respond to a visual target while simultaneously monitoring an auditory stream for a predefined auditory target. *Alternating attention* was assessed through a computerized non-verbal cancellation task that required children to flexibly shift attentional focus between changing stimulus sets, with cancellation demands first involving letters and subsequently visual–spatial symbols. *Reaction times* were measured under two conditions. *Simple reaction time* indexed basic alertness and readiness to respond to neutral, unselected stimuli. The stimuli are presented at the center of the screen and are preceded by a cue stimulus to direct fixation. After the visual stimulus appears, the child is instructed to press a specific computer key. *Reaction time related to a choice* required stimulus discrimination and response selection among multiple alternatives, with children pressing the key corresponding to the target stimulus within a set of stimuli. Unlike simple reaction time tasks, this condition entails the selective processing of relevant information and the inhibition of competing stimuli, thereby capturing an additional component of *selective attention*. *Short-term attentional span* was assessed using a computerized digit span task comprising forward and backward conditions. The total score, calculated as the sum of the scores obtained in the two conditions, was used as the overall index of attentional span.

Stimulus presentation and response recording were fully automated, ensuring standardized administration across participants. For all tasks, performance indices included accuracy and response times expressed in seconds. The battery showed good to excellent psychometric properties, with test–retest reliability coefficients ranging from 0.82 to 0.92 and concurrent validity indices between 0.80 and 0.90.

Children’s social functioning profiles were assessed using a *Q-sort methodology*. This approach describes children’s typical behavioral patterns during everyday interactions with non-parental caregivers based on systematic observation.

The Q-sort procedure focused on observable behaviors displayed by children during routine preschool activities, including responsiveness to the educator, cooperation, emotional expressiveness, and behavioral organization within educator-mediated interactions. Observations were conducted in natural classroom contexts while educators followed their usual routines. Each child’s behavior was described by sorting a predefined set of 90 items according to how characteristic each behavior was of the child. Following standard Q-sort procedures, items were sorted into nine categories ranging from “least characteristic” to “most characteristic,” using a forced distribution. Each child’s sort was then correlated with a criterion profile representing an optimally adaptive pattern of social functioning within educator-child relationships. The resulting Pearson correlation coefficient served as a continuous index of social functioning, with higher values indicating more adaptive, organized, and responsive behavioral profiles. This continuous Q-sort social functioning score was then categorized following established conventions in Q-sort research (Ahnert et al., 2006).

In the present study, internal consistency of the social functioning profile scores was adequate (Cronbach’s *α* ≈ 0.80). Inter-observer agreement, calculated on a subsample of observations, was also satisfactory (*r* ≈ 0.75), in line with values reported in prior studies employing Q-sort methodologies in educational settings (Van Ijzendoorn et al., 2004).

Children’s ability to learn was assessed through an observational questionnaire designed to provide a global measure of learning-related functioning in preschool-aged children.

The instrument provides a global composite score reflecting children’s general learning abilities, as well as domain-specific scores capturing distinct but interrelated skill areas involved in early learning. Specifically, the questionnaire includes items assessing general learning-related abilities, including *behavioral skills* (adaptability to change, cooperation, and autonomy), *motor skills* (fine and gross motor coordination), *linguistic comprehension* (listening comprehension, understanding of verbal instructions, and conversational skills), *oral production* (clarity of expression, narrative abilities, morphosyntactic complexity, and lexical richness), *metacognitive skills* (intentional use of learning strategies, monitoring of cognitive processes, and awareness of misunderstanding), and *other cognitive abilities* (memory, visual-motor coordination, and spatial orientation). In addition to general learning abilities, the questionnaire assesses specific abilities related to early school learning, including *prerequisites for literacy* (phonological and phonemic skills, grapheme discrimination, sequential processing of phonemes, and integration of oral and written language) and *prerequisites for mathematics* (symbol-number associations and magnitude discrimination).

The measure was completed following a period of systematic observation during regular preschool activities, in accordance with standardized administration procedures. Both global scores and domain-specific scores were derived and included in the statistical analyses.

In the present sample, the questionnaire demonstrated good internal consistency for the global ability to learn score (Cronbach’s *α* = 0.84), with domain-specific reliability coefficients ranging from acceptable to good (*α*s between 0.71 and 0.82). Inter-rater agreement, calculated on a subsample of children, was satisfactory (*r* = 0.73, *p* < 0.01). Overall, these indices support the reliability and psychometric adequacy of the observational measure for assessing learning-related abilities in preschool-aged children.

### 2.3 Procedure

To maintain ecological validity and minimize disruption to the natural educational setting, all data collection activities were conducted during the morning hours while children were engaged in their usual preschool routines. The Attention and Concentration Battery (Di Nuovo, 2000) was administered individually in a quiet and familiar space within the preschool environment. Each child completed the battery under the supervision of a trained researcher. Prior to each subtest, a brief practice session was provided to ensure task comprehension, regardless of children’s previous experience with computerized assessments. All tasks were counterbalanced across participants to reduce potential order effects.

Observational data for the assessment of social functioning profiles and learning abilities were collected by a trained observer with expertise in developmental psychology and early childhood assessment. Before data collection, the observer completed a training phase focused on standardizing observation procedures and coding criteria, in order to ensure methodological consistency and reliability.

Children were observed during routine classroom activities while educators continued their standard practices, allowing the assessment to capture typical interactional behaviors. The order of administration of the attention battery and the observational protocols was randomized across participants to reduce potential order effects, and observations were distributed across multiple sessions to enhance the representativeness of the data.

The study was conducted in accordance with the ethical principles outlined in the Declaration of Helsinki, the Code of Ethics of Italian Psychologists (Law No. 56, February 18, 1989), the Italian Data Protection Law (Legislative Decree 196/2003), and the Code of Ethics for Psychological Research (March 27, 2015), as approved by the Italian Association of Psychology. The Internal Ethic Review Board of Psychology Research of the Department of Educational Sciences of the University of Catania approved the research protocol (prot. Ierb-Edunict-2024.03.07/02). Written informed consent was obtained from parents or legal guardians before participation.

### 2.4 Statistical analyses

A sensitivity power analysis was conducted using G*Power 3.1 (Faul et al., 2009) to determine the minimum effect size detectable with the available sample size. With *N* = 139, *α* = 0.05, power = 0.80, and a maximum of 16 predictors (including aspects of attention, the continuous index of social functioning, demographic covariates, and selected interaction terms), the minimum detectable effect size was *f*^2^ = 0.15. This indicates that the study was adequately powered to detect medium-sized effects in the most complex hierarchical regression models estimated. The following sections describe the data preparation procedures and analytical strategy adopted.

Prior to analysis, all continuous variables were transformed into standardized z-scores. This procedure ensured comparability across indices derived from different measurement scales, including response times and accuracy scores, and facilitated interpretation of regression coefficients and interaction terms. Standardization was applied to all attentional indices, learning ability scores (global and domain-specific), and the continuous measure of social functioning profiles in the educator-child relationship. The distribution of all continuous variables was inspected to assess normality prior to analysis. Given the moderate sample size, skewness and kurtosis values were used as indicators of distributional adequacy, following the guidelines of West et al. (1995), who recommend thresholds of |2| for skewness and |7| for kurtosis as criteria for significant deviation from normality. All study variables fell well within these thresholds, supporting the assumption of approximate normality. Levene’s tests confirmed homogeneity of variances across the three social functioning groups for all variables examined. The use of parametric tests was therefore considered appropriate (Field, 2018; Tabachnick and Fidell, 2019).

For attentional tasks providing both accuracy (proportion of correct responses) and response time indices, the Balanced Integration Score (BIS) was computed following Liesefeld et al. (2015). The BIS integrates speed and accuracy by subtracting standardized response time from standardized accuracy (BIS = zPC − zRT), thereby reducing distortions associated with speed–accuracy trade-offs and providing a more robust index of attentional efficiency. BIS scores were calculated for each relevant task across the full sample to preserve interindividual variability. The BIS was not computed for the Digit Span task, which is indexed exclusively by accuracy.

Following these preliminary data preparation steps, group comparisons were conducted using independent-samples *t*-tests to examine differences by gender and age group. To provide a complementary descriptive characterization of the sample, children were additionally divided into three groups based on percentile thresholds of the continuous social functioning score (low, medium, high social functioning). This trichotomization was adopted exclusively for descriptive purposes and was not used in the inferential analyses. One-way ANOVAs with Tukey *post hoc* tests were performed to examine differences across these three groups in attentional and learning variables, with the aim of illustrating the pattern of variability associated with different levels of social functioning prior to the moderation analyses. All inferential analyses were conducted using the continuous social functioning score, consistent with standard recommendations for moderation testing that caution against the unnecessary dichotomization or trichotomization of continuous variables (Aiken et al., 1991; Hayes, 2022; MacCallum et al., 2002).

To account for the multidimensional structure of attention, hierarchical multiple regression models were subsequently estimated using ordinary least squares estimation. In all models, age and gender were entered in the first block. A two-stage modeling strategy was planned prior to analysis to address a well-recognized challenge in multivariable regression with correlated predictors: shared variance among attentional components can attenuate individual coefficients in full-model specifications, making it difficult to identify which components carry meaningful independent associations (Harrell, 2015). In the first stage, comprehensive models including all measured attentional components simultaneously were estimated for each learning outcome, alongside social functioning and demographic covariates, to evaluate the full predictor space without *a priori* exclusions. Collinearity diagnostics (VIF and tolerance values) were examined to ensure that multicollinearity did not bias parameter estimates. In the second stage, reduced parsimonious models were estimated retaining attentional components showing statistically significant (*p* < 0.05) or trend-level (*p* < 0.10) associations in the first-stage models. The inclusion of trend-level predictors was intentional: in multivariable frameworks with correlated predictors, a rigid significance threshold risks excluding components whose associations are genuine but attenuated by shared variance (Harrell, 2015). This two-stage strategy follows established methodological recommendations for variable selection in multivariable regression (Babyak, 2004; Harrell, 2015) and reduces overfitting risk relative to a single broad-inclusion model, as the final models on which inference is based contain fewer predictors relative to sample size.

Moderation analyses were conducted within the hierarchical regression framework to test whether the association between different aspects of attention and learning outcomes varied as a function of children’s social functioning profiles. Interaction terms were introduced in Block 3 for predictors demonstrating independent main effects in Block 2, consistent with standard guidelines for moderation testing in hierarchical regression (Aiken et al., 1991; Hayes, 2022). The continuous social functioning score was mean-centered prior to computing interaction terms. Models were structured in three steps: (1) covariates, (2) main effects of attention aspects and social functioning, and (3) interaction terms (Attention × Social Functioning). The incremental contribution of interaction effects was evaluated through changes in *R*^2^ and associated *F*-tests. To facilitate comparison across models and provide a complete picture of effect magnitude, Cohen’s *f*^2^ was computed as an incremental effect size index for Block 2 and Block 3 of each hierarchical regression model, following the formula *f*^2^ = Δ*R*^2^/(1 − *R*^2^), where *R*^2^ refers to the variance explained by the full model—that is, the *R*^2^ of the most complete model specification including all blocks (Cohen, 1988). Following established conventions, *f*^2^ values of 0.02, 0.15, and 0.35 were used as benchmarks for small, medium, and large effects, respectively.

When significant interactions emerged, conditional effects were probed by estimating simple slopes at low (−1 SD), mean, and high (+1 SD) levels of social functioning and by identifying Johnson–Neyman regions of significance. Conditional effects were computed using the PROCESS macro (Version 5.0). All analyses were conducted using IBM SPSS Statistics (Version 30.0).

## 3 Results

### 3.1 Descriptive statistics and preliminary group comparisons

Descriptive statistics for all study variables are reported in Supplementary Table 1. Means and standard deviations are presented using raw scores for social functioning and learning outcomes, and separate indices of response time and accuracy for attentional tasks to preserve interpretability of performance metrics. For inferential analyses, attentional performance was operationalized using the Balanced Integration Score (BIS), which integrates standardized accuracy and response time indices into a single measure of attentional efficiency.

Preliminary analyses were conducted to descriptively characterize the sample with respect to gender, age group, and social functioning profiles. Females showed higher social functioning profile scores than males (*p* < 0.001). Gender differences also emerged in specific aspects of attention, including simple reaction time (*p* < 0.01) and digit span (*p* < 0.05), whereas no statistically significant differences were observed across the learning ability indices.

Age-related differences followed a consistent developmental pattern. Five-year-old children outperformed four-year-olds across multiple aspects of attention, including auditory focused attention, visual focused attention, visual–spatial focused attention, digit span, divided attention, and both verbal and visual–spatial alternating attention (all *p*s < 0.001). Age differences were likewise observed across all learning ability indices (all *p*s < 0.001), reflecting developmental variability within the preschool period.

To provide a complementary descriptive perspective aligned with the moderation analyses, the sample was divided into three percentile-based groups of social functioning profiles (low, medium, high). One-way ANOVAs indicated significant group differences in simple reaction time (*p* = 0.02), reaction time related to a choice (*p* < 0.001), and visual–spatial focused attention (*p* < 0.001). *Post hoc* comparisons indicated that children in the high social functioning group performed significantly better than those in the low group in both simple reaction time and visual–spatial focused attention (*p*s ≤ 0.01). For reaction time related to a choice, a graded pattern emerged, with performance progressively increasing from low to medium (*p* < 0.001) and from medium to high (*p* = 0.01) social functioning.

Across all learning indices, significant group differences were observed (*p*s ≤ 0.001). Post hoc analyses consistently showed that children in the low social functioning group scored significantly lower than those in the medium and high groups across global learning ability and domain-specific indices (*p*s = 0.001–0.002). Overall, these patterns underscore the contribution of social functioning profiles as a meaningful source of variability in early learning performance, highlighting their relevance not only for relational adjustment but also for learning outcomes during the preschool years.

### 3.2 Moderation analyses

Hierarchical multiple regression models were estimated to examine whether children’s social functioning profiles moderated the associations between components of attention and learning outcomes, following the analytical strategy described in Section 2.4. Collinearity diagnostics indicated no concerns (all VIF < 4; tolerance > 0.25). To support evaluation of robustness, coefficients for the Divided Attention × Social Functioning interaction term extracted from the full hierarchical models—including all attentional predictors simultaneously in Block 2, before the reduction stage, with interaction terms introduced in Block 3—are reported in Supplementary Table 2. Results are consistent across both model specifications, confirming that the core finding is not an artifact of the model selection procedure.

#### 3.2.1 Moderation effects on general learning abilities

In the comprehensive models including all attention scores, visual focused attention, visual–spatial focused attention, divided attention, and digit span showed significant independent associations with general learning abilities (all *p*s < 0.05) and were retained in subsequent analyses.

After controlling for age and gender, visual focused attention (*p* = 0.01), visual–spatial focused attention (*p* = 0.01), and digit span (*p* = 0.04) showed significant positive associations with general learning abilities. In addition, a significant Divided Attention × Social Functioning interaction was observed (*p* = 0.02), indicating that the contribution of divided attention to general learning abilities varied across levels of social functioning. Detailed regression coefficients and model statistics are reported in Table 1. The incremental contribution of Block 2 was medium-to-large (*f*^2^ = 0.36), and the contribution of Block 3 was small (*f*^2^ = 0.09).

**Table 1:** Hierarchical regression predicting general learning abilities.

Predictor	*B*	*SE*	*β*	*t*	*p*
Block 1: covariates					
Age	1.12	0.14	0.57	7.9	<0.001
Sex	0.09	0.14	0.05	0.65	0.52
Model *R*^2^ = 0.32					
Block 2: main effects					
Age	0.37	0.19	0.18	1.85	0.06
Sex	0.15	0.13	0.07	1.10	0.27
Social functioning	0.08	0.07	0.08	1.25	0.21
Visual focused attention	0.12	0.05	0.20	2.50	0.01
Visual–spatial focused attention	0.12	0.04	0.21	2.77	0.006
Divided attention	0.10	0.06	0.14	1.81	0.05
Digit span	0.19	0.09	0.19	2.01	0.04
Model *R*^2^ = 0.49, Δ*R*^2^ = 0.17, *f*^2^ = 0.36					
Block 3: interaction terms					
Age	0.40	0.20	0.20	1.99	0.05
Sex	0.10	0.13	0.05	0.78	0.43
Social functioning	0.12	0.07	0.12	1.69	0.09
Visual focused attention	0.14	0.05	0.23	2.62	0.01
Visual–spatial focused attention	0.116	0.045	0.203	2.586	0.01
Divided attention	0.09	0.06	0.13	1.68	0.09
Digit span	0.19	0.09	0.19	2.00	0.04
Visual focused attention × Social functioning	0.05	0.04	0.11	1.33	0.18
Visual–spatial focused attention × Social functioning	0.06	0.04	0.10	1.47	0.14
Divided attention × Social functioning	0.14	0.06	0.18	2.21	0.02
Digit span × Social functioning	0.15	0.08	0.15	1.89	0.06
Model *R*^2^ = 0.53, Δ*R*^2^ = 0.04, *f*^2^ = 0.09					

All continuous predictors were standardized prior to analysis. Age and gender were entered as covariates in Block 1. Interaction terms were computed using mean-centered predictors. Δ*R*^2^ reflects change from the previous block. *f*^2^ = Cohen’s incremental effect size, calculated as Δ*R*^2^/(1 − *R*^2^). Following Cohen (1988), values of 0.02, 0.15, and 0.35 indicate small, medium, and large effects, respectively.

**Figure 1:**
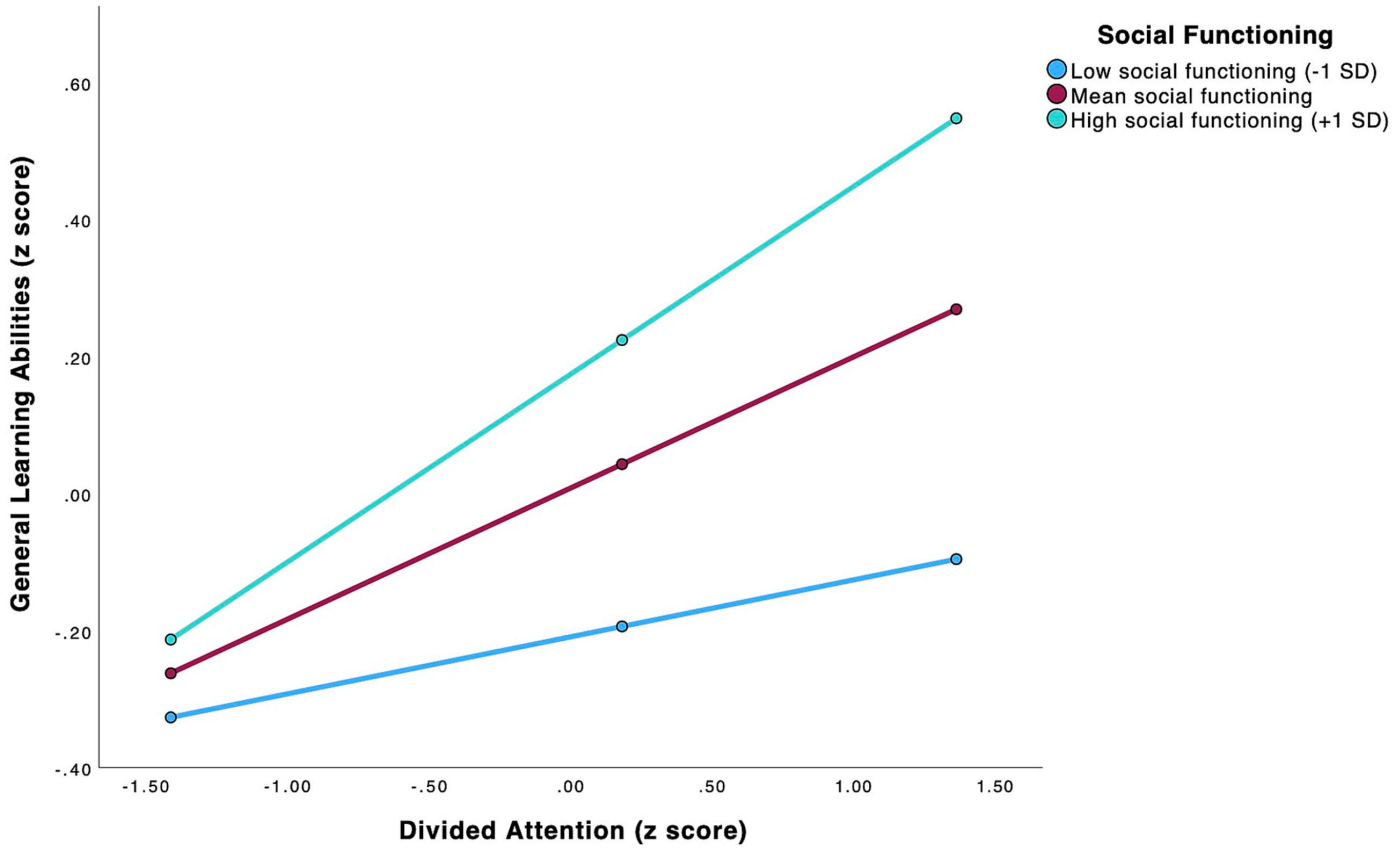
Simple slopes illustrating the interaction between divided attention and social functioning profiles in predicting general learning abilities.

Simple slopes analyses clarified the direction of this interaction (Figure 1). At mean levels of social functioning, divided attention was positively associated with general learning abilities (*p* = 0.04), and this association was stronger at high levels of social functioning (*p* = 0.01). The Johnson–Neyman analysis indicated that the effect of divided attention became statistically significant at values of social functioning slightly above the sample mean.

#### 3.2.2 Moderation effects on prerequisites for literacy

To identify the components of attention to be retained in the hierarchical moderation models, a preliminary regression including all measured components of attention and social functioning was conducted. Visual–spatial focused attention (*p* = 0.01), divided attention (*p* = 0.02), visual–spatial alternating attention (*p* = 0.04), and digit span (*p* < 0.001) showed significant independent associations and were therefore retained in subsequent models.

Controlling for age and sex, digit span (*p* = 0.01), visual–spatial focused attention (*p* = 0.02), and divided attention (*p* = 0.005) showed significant associations with prerequisites for literacy in the final model. Among the interaction terms, the Divided Attention × Social Functioning interaction was statistically significant (*p* = 0.007). Detailed coefficients are reported in Table 2. The incremental contribution of Block 2 was medium-to-large (*f*^2^ = 0.33), and the contribution of Block 3 was small (*f*^2^ = 0.07).

**Table 2:** Hierarchical regression predicting prerequisites for literacy.

Predictor	*B*	*SE*	*β*	*t*	*p*
Block 1: covariates					
Age	1.18	0.14	0.59	8.57	<0.001
Sex	0.13	0.14	0.07	0.95	0.34
Model *R*^2^ = 0.35					
Block 2: main effects					
Age	0.37	0.20	0.18	1.83	0.07
Sex	0.15	0.14	0.08	1.12	0.27
Social functioning	0.12	0.07	0.12	1.79	0.07
Digit span	0.20	0.09	0.20	2.19	0.03
Visual–spatial focused attention	0.11	0.04	0.19	2.57	0.01
Divided attention	0.14	0.05	0.19	2.61	0.01
Visual–spatial alternating attention	0.13	0.07	0.15	1.84	0.06
Model *R*^2^ = 0.51, Δ*R*^2^ = 0.15, *f*^2^ = 0.33					
Block 3: interaction terms					
Age	0.33	0.20	0.16	1.61	0.11
Sex	0.14	0.13	0.07	1.01	0.31
Social functioning	0.14	0.07	0.14	1.93	0.05
Digit span	0.23	0.09	0.23	2.48	0.01
Visual–spatial focused attention	0.11	0.04	0.19	2.42	0.02
Divided attention	0.15	0.05	0.20	2.84	0.005
Visual–spatial alternating attention	0.12	0.07	0.14	1.78	0.08
Digit span × Social functioning	0.11	0.08	0.11	1.43	0.15
Visual–spatial focused attention × Social functioning	0.05	0.04	0.09	1.27	0.21
Divided attention × Social functioning	0.17	0.06	0.22	2.73	0.007
Visual–spatial alternating attention × Social functioning	0.03	0.06	0.04	0.49	0.63
Model *R*^2^ = 0.54, Δ*R*^2^ = 0.03, *f*^2^ = 0.07					

All continuous predictors were standardized prior to analysis. Age and sex were entered as covariates in Block 1. Interaction terms were computed using mean-centered predictors. Δ*R*^2^ reflects change from the previous block. *f*^2^ = Cohen’s incremental effect size, calculated as Δ*R*^2^/(1 − *R*^2^). Following Cohen (1988), values of 0.02, 0.15, and 0.35 indicate small, medium, and large effects, respectively.

**Figure 2:**
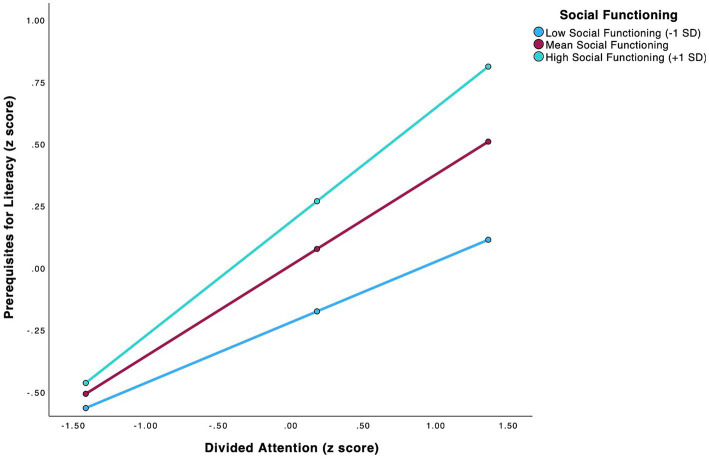
Simple slopes illustrating the interaction between divided attention and social functioning profiles in predicting prerequisites for literacy.

Simple slopes analyses clarified this interaction (Figure 2). At low levels of social functioning (−1 SD), divided attention was not associated with literacy prerequisites (*p* > 0.05). At mean levels, the association was positive and significant (*p* = 0.04), and it was stronger at high levels (*p* < 0.001). Johnson–Neyman analysis indicated that the effect became significant at values of social functioning slightly above the sample mean.

#### 3.2.3 Moderation effects on prerequisites for mathematics

A preliminary regression including all components of attention identified visual–spatial focused attention as showing a significant independent association (*p* = 0.02), while visual focused attention (*p* = 0.048), divided attention (*p* = 0.049), and digit span (*p* = 0.051) showed trend-level associations. These components were retained in the hierarchical moderation models.

In the final model, visual focused attention (*p* = 0.02), visual–spatial focused attention (*p* = 0.03), divided attention (*p* = 0.04), and digit span (*p* = 0.03) showed significant positive associations with prerequisites for mathematics. The Divided Attention × Social Functioning interaction was statistically significant (*p* = 0.01). Full regression results are reported in Table 3. The incremental contribution of Block 2 was large (*f*^2^ = 0.35), and the contribution of Block 3 was small (*f*^2^ = 0.09).

**Table 3:** Hierarchical regression predicting prerequisites for mathematics.

Predictor	*B*	*SE*	*β*	*t*	*p*
Block 1: covariates					
Age	1.18	0.14	0.59	8.59	<0.001
Sex	0.13	0.14	0.07	0.96	0.34
Model *R*^2^ = 0.36					
Block 2: main effects					
Age	0.44	0.19	0.22	2.28	0.02
Sex	0.18	0.13	0.09	1.32	0.19
Social functioning	0.10	0.07	0.10	1.52	0.13
Visual focused attention	0.10	0.05	0.17	2.27	0.02
Visual–spatial focused attention	0.10	0.04	0.18	2.47	0.01
Divided attention	0.11	0.06	0.16	2.07	0.04
Digit span	0.19	0.09	0.19	2.05	0.04
Model *R*^2^ = 0.51, Δ*R*^2^ = 0.16, *f*^2^ = 0.35					
Block 3: interaction terms					
Age	0.46	0.19	0.23	2.37	0.02
Sex	0.14	0.13	0.07	1.04	0.30
Social functioning	0.14	0.07	0.14	1.97	0.05
Visual focused attention	0.12	0.05	0.20	2.36	0.02
Visual–spatial focused attention	0.09	0.04	0.17	2.20	0.03
Divided attention	0.11	0.06	0.15	2.01	0.04
Digit span	0.21	0.09	0.21	2.19	0.03
Visual focused attention × Social functioning	0.04	0.04	0.09	1.17	0.25
Visual–spatial focused attention × Social functioning	0.06	0.04	0.11	1.64	0.10
Divided attention × Social functioning	0.15	0.06	0.19	2.48	0.01
Digit span × Social functioning	0.13	0.08	0.13	1.72	0.08
Model *R*^2^ = 0.55, Δ*R*^2^ = 0.04, *f*^2^ = 0.09					

All continuous predictors were standardized prior to analysis. Age and gender were entered as covariates in Block 1. Interaction terms were computed using mean-centered predictors. Δ*R*^2^ reflects change from the previous block. *f*^2^ = Cohen’s incremental effect size, calculated as Δ*R*^2^/(1 − *R*^2^). Following Cohen (1988), values of 0.02, 0.15, and 0.35 indicate small, medium, and large effects, respectively.

**Figure 3:**
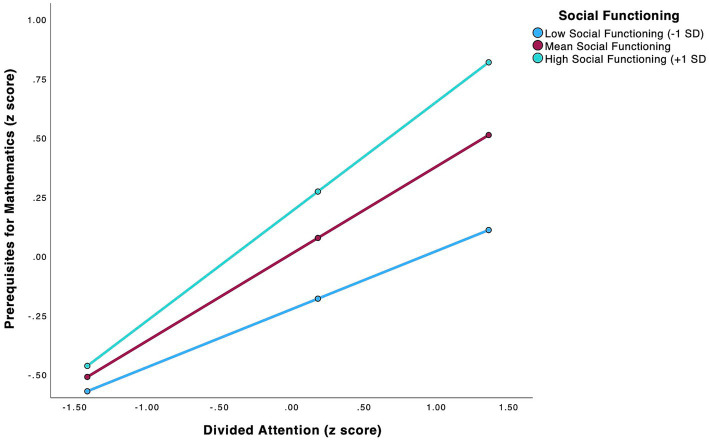
Simple slopes illustrating the interaction between divided attention and social functioning profiles in predicting prerequisites for mathematics.

Simple slopes analyses (Figure 3) showed that divided attention was not significantly associated with mathematics prerequisites at low levels of social functioning (*p* > 0.05), whereas the association was positive and statistically significant at mean and high levels (*p* < 0.01). Johnson–Neyman analysis indicated that the conditional effect of divided attention became statistically significant at values of social functioning slightly above the sample mean.

#### 3.2.4 Moderation effects across specific learning domains

Across specific learning domains (behavioral skills, motor skills, linguistic comprehension, oral production, metacognitive skills, and other cognitive abilities), the hierarchical regression models indicated highly consistent patterns of association (see Table 4). The inclusion of interaction terms in Block 3 produced small but systematic increments in explained variance (Δ*R*^2^ ranging from 0.033 to 0.037; *f*^2^ is ranging from 0.07 to 0.10). The Divided Attention × Social Functioning interaction was statistically significant across all domains (*p*s ≤ 0.019), with standardized coefficients ranging from 0.187 to 0.197. Although the increase in explained variance for metacognitive skills did not reach conventional statistical significance at the model level (*p* = 0.069), the interaction term remained statistically significant. No other interaction terms were significant across domains.

**Table 4:** Hierarchical regression models predicting specific learning domains: interaction effects.

Learning domain	*R*^2^ (Block 3)	Δ*R*^2^ (Block 3)	*p* Δ*R*^2^	*β* divided × social	*p* interaction	*f* ^2^
Behavior	0.545	0.036	0.045	0.196	0.013	0.08
Motor skills	0.543	0.036	0.048	0.195	0.014	0.08
Linguistic comprehension	0.545	0.036	0.045	0.196	0.013	0.08
Oral production	0.545	0.036	0.046	0.195	0.014	0.10
Metacognition	0.538	0.033	0.069	0.187	0.019	0.07
Other cognitive abilities	0.549	0.037	0.040	0.197	0.013	0.08

*β* values refer to standardized coefficients from Block 3. Δ*R*^2^ indicates the increment in explained variance attributable to the inclusion of interaction terms. *f*^2^ = Cohen’s incremental effect size for Block 3 [Δ*R*^2^/(1 − *R*^2^_full)]; 0.02 = small, 0.15 = medium, 0.35 = large (Cohen, 1988).

## 4 Discussion

The present study was designed to examine how distinct components of attention contribute to early learning across different levels of specificity and, most critically, whether their functional impact varies across different social functioning profiles during the preschool years. While the findings confirm that attention operates as a multifaceted system composed of functionally distinct aspects, consistent with neurocognitive and developmental models of attentional networks (Petersen and Posner, 2012; Posner and Rothbart, 2014; Rueda, 2014), the core contribution of the study lies in demonstrating that the association between specific aspects of attention and learning outcomes varies systematically as a function of children’s social functioning profiles, suggesting that the association between attentional capacities and observable learning competence is socially embedded rather than purely cognitive (Commodari, 2013a).

Preliminary analyses further confirmed the relevance of social functioning profiles as a meaningful dimension of variability in early development. Children characterized by more adaptive social functioning profiles showed systematically stronger performance across global and domain-specific learning indices and in several aspects of attention, indicating that differences in social functioning are already reflected in both cognitive and learning-related functioning during the preschool years. In addition, consistent age-related differences were observed across attentional and learning abilities, in line with developmental expectations, whereas gender differences were limited to social functioning profiles and specific aspects of attention and did not extend to learning outcomes. Together, these findings provide an important contextual framework for interpreting the subsequent conditional analyses.

At the most global level, general learning abilities showed consistent positive associations with multiple aspects of attention, including visual focused attention, visual–spatial focused attention, and short-term attentional span, which appear as stable correlates of broad learning competence in early childhood. In particular, visual-focused attention and visual–spatial focused attention both showed stable associations with overall learning competence. These results underscore that general learning ability in early childhood is largely related to the child’s capacity to selectively concentrate on task-relevant visual information and to the selective processing of spatially organized input in structured learning situations, where symbolic materials and task-relevant cues are predominantly organized in visual and spatial formats (Posner and Rothbart, 2014). Moreover, these findings are consistent with a role of short-term attentional span in early learning, suggesting that the ability to temporarily maintain information during goal-directed activities is associated with stronger learning outcomes (Lanyu, 2024). Presumably, the span of attention supports the capacity to sustain internal representations across sequential instructions and multi-step activities.

When moving from general abilities to more specific learning prerequisites, partially differentiated attentional configurations emerged. Visual focused attention, visual–spatial focused attention, and visual–spatial alternating attention showed significant associations with prerequisites for literacy, consistent with the visually organized and spatially structured nature of early symbolic processing (Commodari, 2017). The domain-specific contribution of visual–spatial alternating attention to literacy prerequisites warrants particular attention. Alternating attention refers to the capacity to flexibly shift attentional focus between different tasks or cognitive sets (Rueda, 2014), and its selective association with literacy prerequisites but not with general learning abilities or mathematics prerequisites reflects the particular demands of early reading acquisition. Emergent literacy skills require children to rapidly and repeatedly transition between visual symbol recognition, phonological representations, and externally provided linguistic cues, a process that places specific demands on the capacity for flexible attentional reorientation across representational systems (Commodari, 2017; Franceschini et al., 2012). By contrast, prerequisites for mathematics and general learning abilities rely more heavily on the processing of spatially organized input and the maintenance of sequential representations, demands that are better served by focused attention and short-term attentional span than by flexible shifting. Therefore, the domain-specific pattern observed for alternating attention reflects a theoretically coherent differentiation in the attentional demands underlying early literacy and mathematics acquisition. Visual-focused attention and visual–spatial focused attention also demonstrated stable associations with prerequisites for mathematics, consistent with the structured processing of spatial-symbolic relations and the coordination of ordered problem-solving routines. A comparable pattern emerged across additional learning domains, including linguistic comprehension, oral production, behavioral functioning, motor skills, metacognition, and other cognitive abilities.

By contrast, divided attention displayed a qualitatively distinct pattern. Although independently associated with learning outcomes, its strength of association varied systematically across social functioning profiles. This graded pattern is theoretically central and can be interpreted within an integrated framework drawing on developmental systems theory and self-regulation research. Divided attention refers to the capacity to coordinate multiple concurrent streams of incoming information simultaneously (MacKay-Brandt, 2011; Song, 2019). In structured educational contexts, this capacity operates within a relational field: children must integrate task-relevant stimuli, verbal instructions from adult caregivers, and ongoing interpersonal signals while sustaining goal-directed engagement. This concurrent coordination is not reducible to internal cognitive efficiency alone. From a developmental systems perspective (Kärtner and Köster, 2024; Thelen and Smith, 1994), cognitive capacities are dynamically expressed within the contextual structures that constitute children’s developmental environments. Social functioning profiles within educator-child relationships constitute precisely such a structure. When these profiles are characterized by greater responsiveness, coherence, and behavioral organization, they may provide the external regulatory scaffolding associated with more effective deployment of attentional resources across competing informational demands. Self-regulation theory offers a complementary mechanistic account: the development of effortful control (the volitional allocation and modulation of attention in goal-directed activity) is scaffolded by the predictability and quality of adult-child interactions (Blair and Raver, 2015; Raver et al., 2012). Children whose interactions with educators are characterized by greater organization and responsiveness may be better positioned to recruit and sustain divided attentional resources during structured learning activities, because the relational context itself reduces the interpretive load associated with concurrent social and task demands. Under less adaptive social functioning profiles, the reduced coherence of interactional cues may impose competing interpretive demands that interfere with simultaneous task processing, thereby attenuating the observed association between divided attention and learning performance.

The specificity of this moderation effect, limited to divided attention and not observed for focused attention or attentional span, reflects the distinct relational sensitivity of this component. Focused attention and short-term attentional span primarily index the efficiency of internal processing resources and their deployment in response to clearly structured, unidimensional stimulus environments. These capacities are less dependent on the coordination of concurrent interpersonal and task demands, and their functional expression is therefore less contingent on the broader social configuration within which learning unfolds.

Against this consistent moderating pattern, visual focused attention, visual–spatial focused attention, and short-term span of attention showed stable associations with performance that did not vary across levels of social functioning. Rather than weakening the overall model, this specificity strengthens its interpretive value. It indicates that social functioning profiles do not exert a diffuse influence across the entire attentional system but are selectively associated with differences in the expression of divided attention across learning outcomes. The increments in explained variance attributable to the interaction terms are modest in absolute terms and should be interpreted accordingly. These values are, however, consistent with effect sizes typically observed for moderation effects in field-based developmental research, where the variance of interaction terms is structurally constrained relative to experimental designs (Frazier et al., 2004; McClelland and Judd, 1993). The practical significance of the present findings therefore does not lie in the magnitude of variance explained by the interaction alone, but in the consistency of the moderation pattern across multiple learning outcomes and its theoretical interpretability within a developmental systems framework. In practical terms, the findings suggest that social functioning profiles represent one among several contextual factors that may be associated with differences in how divided attentional resources relate to learning performance, rather than a dominant determinant of learning competence.

Taken together, these findings indicate that social functioning profiles do not independently determine learning outcomes but are concurrently associated with differences in how a specific component of the attentional system—divided attention—relates to learning performance across multiple levels of specificity. The observed pattern suggests that the association between attentional capacities and learning outcomes varies as a function of the social configurations within which children engage in adult-guided activities (Mahone and Schneider, 2012; Yussen, 1974). Given the cross-sectional design, these patterns reflect concurrent associations and do not permit conclusions about developmental trajectories or causal directionality.

### 4.1 Strengths and limitations

The present study is characterized by several methodological strengths. The use of a multi-method approach, integrating computerized assessments of attentional functioning with standardized observational measures of social functioning and learning outcomes in naturalistic classroom contexts, enhances ecological validity and captures behavior as it unfolds within everyday educational activities. The inclusion of a continuous index of social functioning in all inferential analyses, combined with a well-validated observational procedure, provides a sensitive and contextually grounded representation of children’s relational behavioral organization within educator–child interactions. Furthermore, observational data were collected by a trained observer and inter-observer agreement, estimated on a subsample, was satisfactory for both social functioning profiles and learning outcomes, supporting the reliability of the measures.

At the same time, some limitations should be considered. The cross-sectional design is appropriate for examining concurrent associations at a developmentally meaningful stage, which aligns with the study’s objectives, as it did not aim to investigate longitudinal trajectories or developmental sequences. Since this design precludes conclusions about temporal ordering and causal directionality, future longitudinal research could test whether the identified associations remain stable, strengthen, or attenuate as attentional systems mature and children transition into formal schooling. Such designs should also incorporate a broader set of individual and contextual variables as potential confounders, to further improve the precision and generalizability of the findings.

### 4.2 Study implications

The present findings carry implications at both theoretical and practical levels. From a broader developmental perspective, they suggest that attentional functioning in early learning should not be conceptualized solely as an individual cognitive characteristic, but as a capacity whose functional relevance emerges within specific relational and social configurations. This perspective also contributes to ongoing efforts to refine the conceptualization of children’s relational functioning in early educational contexts. Social functioning profiles as assessed in the present study capture observable patterns of behavioral organization within educator-child interactions that draw on, but are not reducible to, established constructs such as attachment security, temperament, or self-regulation. The Q-sort observational procedure employed here is specifically sensitive to the relational-behavioral level of analysis, capturing how children organize their cooperation, emotional availability, and responsiveness within a specific adult-guided context, rather than the quality of the affective bond, constitutionally based reactivity, or individual neurobehavioral regulatory capacity. The finding that social functioning profiles selectively moderate the association between divided attention and learning outcomes, rather than exerting a diffuse influence across the attentional system, is consistent with this conceptualization: what appears to condition the association between divided attention and observable learning performance is not a broad dispositional characteristic of the child, but the specific relational organization within which attentional resources are concurrently deployed during adult-guided activities.

The pattern of results also clarifies the differential role of distinct components of attention in early learning. The consistent associations observed for visual focused attention, visual–spatial focused attention, and short-term span of attention across learning domains indicate that the ability to selectively concentrate and temporarily maintain information constitutes a foundational attentional infrastructure that supports children’s engagement across diverse interactional contexts, regardless of the relational configuration in which learning unfolds. These capacities appear to sustain children’s participation in a broad range of adult-child exchanges, including exploratory, play-based, and routine interactions, and their functional expression is less contingent on the social ecology of the learning environment. Divided attention, by contrast, displays a context-sensitive pattern and its association with learning outcomes varies as a function of the relational coherence and interactional alignment that social functioning profiles provide. During the preschool years, interactions with non-familial adult caregivers constitute highly salient contexts in which children must simultaneously integrate task demands, verbal guidance, and interpersonal signals. In these contexts, the capacity to coordinate concurrent informational streams does not operate in isolation from the relational structure within which activities unfold, and its functional expression in observable performance appears to be differentially associated with the quality of that structure.

In practical terms, these findings encourage moving beyond a view of attentional functioning as a fixed, context-independent trait. Children’s capacity to manage concurrent informational demands may be differentially expressed across relational environments: structured and predictable interactional contexts may be associated with more effective coordination of simultaneous informational streams, whereas less organized relational configurations may be linked to greater variability in how divided attentional resources relate to observable learning performance. More broadly, the findings reinforce the importance of integrating cognitive and social perspectives when examining early learning processes. Children’s attentional systems provide the internal architecture necessary for engagement, yet their concurrent expression in observable performance is associated with the social configurations that characterize interactions with adult caregivers during a critical developmental period. Supporting alignment between attentional demands and relational organization across early childhood contexts may therefore represent a meaningful avenue for promoting adaptive learning trajectories.

## 5 Conclusion

The present study advances our understanding of early learning by clarifying how distinct aspects of attention contribute to learning abilities during the preschool years and, crucially, how their functional relevance varies across social functioning profiles within interactions with adult caregivers outside the family context. Rather than assuming a uniform association between relational quality and learning outcomes, the findings indicate that some aspects of attention contribute stably across domains, whereas others show context-dependent variability linked to differences in social functioning.

Visual and visual–spatial focused attention, together with short-term attentional span, are consistently associated with general learning abilities and specific learning prerequisites. These aspects of attention appear to provide a relatively stable cognitive infrastructure that supports children’s engagement in structured, adult-guided activities across early childhood contexts. In contrast, divided attention followed a differentiated pattern: its association with learning outcomes strengthened systematically as social functioning profiles became more adaptive. This finding suggests that the capacity to coordinate multiple streams of information simultaneously does not translate into observable competence in a uniform manner but is sensitive to the relational configurations within which activities unfold.

These results support a context-sensitive account of attentional functioning in early childhood, in which social functioning profiles are concurrently associated with differences in the extent to which divided attention relates to observable learning performance. By identifying social functioning profiles as a contextual condition associated with this differential pattern, the study contributes to integrative models that conceptualize early learning as emerging from the interplay between domain-specific cognitive processes and the social environments in which children participate, clarifying how cognitive and social dimensions co-occur during a period of heightened developmental plasticity.

In conclusion, the present work provides a theoretically grounded and empirically supported contribution by demonstrating that early learning competence reflects not only the internal organization of attention, but also the social contexts in which specific aspects of attention are deployed. Understanding this alignment between attention and social functioning profiles represents an important step toward more context-sensitive models of early developmental functioning across adult-guided environments.

## Data Availability

The raw data presented in this study are available from the corresponding authors upon reasonable request.
