# Supplementary material for: Social functioning profiles, attention skills and learning: what relationships in early childhood?
Source: Front Psychol. 2026 Jun 2;17:1819472. doi: 10.3389/fpsyg.2026.1819472 (PMC13268933; doi:10.3389/fpsyg.2026.1819472)
Supplement: Supplementary file 1 [file Table_1.docx]

Supplementary Material

# Supplementary Tables

**Supplementary Table 1**. Descriptive statistics of study variables (N = 139)

| **Measure** | ***M*** | ***SD*** |  |
| --- | --- | --- | --- |
| Social functioning | 0.47 | 0.14 |  |
| *Main aspects of attention* | | | |
| Simple reaction time - Correct | 28.14 | 4.41 |  |
| Simple reaction time - Time | 0.43 | 0.26 |  |
| Reaction time related to a choice - Correct | 7.53 | 9.28 |  |
| Reaction time related to a choice - Time | 0.71 | 0.76 |  |
| Auditory focused attention - Correct | 7.40 | 1.72 |  |
| Auditory focused attention - Time | 0.92 | 0.31 |  |
| Visual focused attention - Correct | 7.73 | 1.86 |  |
| Visual focused attention - Time | 0.75 | 0.22 |  |
| Visual-spatial focused attention - Correct | 8.57 | 2.72 |  |
| Visual-spatial focused attention - Time | 0.63 | 0.21 |  |
| Digit span | 1.16 | 1.60 |  |
| Divided Attention - Correct | 5.52 | 2.62 |  |
| Divided Attention - Time | 0.93 | 0.41 |  |
| Verbal alternating attention - Correct | 6.15 | 2.84 |  |
| Verbal alternating attention - Time | 114.01 | 68.61 |  |
| Visual-spatial alternating attention - Correct | 5.65 | 3.07 |  |
| Visual-spatial alternating attention - Time | 125.05 | 70.06 |  |
| *Learning abilities* | | | |
| Behavior | 28.00 | 3.53 |  |
| Motor skills | 6.22 | 0.78 |  |
| Linguistic comprehension | 9.34 | 1.18 |  |
| Oral production | 15.55 | 1.96 |  |
| Metacognition | 12.45 | 1.57 |  |
| Other cognitive abilities | 31.11 | 3.92 |  |
| Prerequisite for literacy | 21.76 | 2.75 |  |
| Prerequisite for mathematics | 9.33 | 1.18 |  |
| General learning abilities | 133.40 | 16.86 |  |

**Supplementary Table 2.** Divided Attention × Social Functioning Interaction: Coefficients from Full Hierarchical Regression Models

| **Learning Outcome** | ***B*** | **SE** | ***β*** | ***t*** | ***p*** |
| --- | --- | --- | --- | --- | --- |
| General learning abilities | .136 | .062 | .176 | 2.213 | .029 |
| Prerequisites for literacy | .167 | .061 | .216 | 2.732 | .007 |
| Prerequisites for mathematics | .149 | .060 | .193 | 2.483 | .014 |

*Note.* Values refer to the unstandardized (B) and standardized (β) coefficients for the Divided Attention × Social Functioning interaction term extracted from the full hierarchical regression models (Block 3), which include all attentional predictors simultaneously in Block 2, before the reduction stage described in the main text. All continuous predictors were standardized prior to analysis. The interaction effect is statistically significant across all three outcomes, consistent with the results reported in Tables 1–3 of the main manuscript.
